# Update on Menopausal Hormone Therapy for Fracture Prevention

**DOI:** 10.1007/s11914-019-00549-3

**Published:** 2019-11-18

**Authors:** Jan J. Stepan, Hana Hruskova, Miloslav Kverka

**Affiliations:** 1 Institute of Rheumatology, Prague, Czech Republic; 2 Department of Obstetrics and Gynecology, First Faculty of Medicine, Prague, Czech Republic; 3 Charles University, Prague, Czech Republic; 4 General University Hospital in Prague, Prague, Czech Republic; 5 Institute of Microbiology of the Czech Academy of Sciences, v.v.i., Prague, Czech Republic; 6 Institute of Experimental Medicine of the Czech Academy of Sciences, v.v.i., Prague, Czech Republic

**Keywords:** Bone cells, Estrogens, Fracture, Postmenopausal osteoporosis, Prevention

## Abstract

**Purpose of Review:**

The goal of the review is to assess the appropriateness of menopausal hormone therapy (MHT) for the primary prevention of bone loss in women at elevated risk in the early years after menopause.

**Recent Findings:**

Estrogen alone or combined with progestin to protect the uterus from cancer significantly reduces the risk of osteoporosis-related fractures. MHT increases type 1 collagen production and osteoblast survival and maintains the equilibrium between bone resorption and bone formation by modulating osteoblast/osteocyte and T cell regulation of osteoclasts. Estrogens have positive effects on muscle and cartilage. Estrogen, but not antiresorptive therapies, can attenuate the inflammatory bone-microenvironment associated with estrogen deficiency. However, already on second year of administration, MHT is associated with excess breast cancer risk, increasing steadily with duration of use.

**Summary:**

MHT should be considered in women with premature estrogen deficiency and increased risk of bone loss and osteoporotic fractures. However, MHT use for the prevention of bone loss is hindered by increase in breast cancer risk even in women younger than 60 years old or who are within 10 years of menopause onset.

## Introduction

Osteoporosis is a common disease characterized by low bone mass, microarchitectural disruption, increased skeletal fragility, decreased bone strength associated with increased fracture risk, and mortality associated with fractures [1]. Due to changes in population demography, the annual number of fragility fractures will rise from 3.5 million in 2010 to 4.5 million in 2025 in the EU [2]. Therapies that are effective in osteoporotic women at high risk of fracture are available. However, to significantly impact total fracture numbers, effective options are needed to prevent early accelerated bone loss in women in the first years after menopause and to delay the necessity of antiresorptive therapies in postmenopausal women as they age. Estrogen deficiency is the major cause of the early postmenopausal increase in bone resorption, bone loss and osteoporosis [3]. Randomized clinical studies have demonstrated that estrogen alone, or combined with progestin to protect the uterus from cancer, reduces the risk of osteoporosis-related fractures. There are currently no clinical guidelines for the management of bone loss in early postmenopausal women. The magnitude of bone remodeling and early bone loss depend on several important genetic factors and bone health characteristics of the women, such as recurrent cycle/ovulatory disturbances; overall nutrition; body mass index; protein, calcium, and vitamin D intakes; physical activity; adequacy of sleep; the psychosocial environment; and cognitive dietary restraint, modulate the bone tissue sensitivity to estrogen deficiency or other factors.

## Antifracture Efficacy of Menopausal Hormone Therapy

According to a meta-analysis of several randomized clinical studies published between 1973 and 2000, as a result of MHT, there was a significant 33% reduction in vertebral fracture [4] and a significant 27% reduction in nonvertebral fractures [5]. The pooled relative risk (RR) of nonvertebral fractures, hip and wrist fractures was significantly reduced in women younger than 60 years [5]. Subsequent double-blind, randomized, placebo-controlled studies have confirmed a significant increase in bone mineral density (BMD) in women on MHT compared with individuals in the placebo group [6–12].

A prospective postmenopausal estrogen/progestin intervention (PEPI) study evaluated the effects of MHT on BMD in 875 healthy postmenopausal women (age 45 to 64 years). After 3 years, women assigned to conjugated equine estrogens (CEE), 0.625 mg/day plus a progestin; or CEE alone gained significantly more BMD in the lumbar spine and proximal femur than those who received placebo [7].

The results of the National Osteoporosis Risk Assessment (NORA) indicated that current but not past hormone use at baseline was associated with a significantly lower risk of osteoporotic fracture in 1 year compared with nonusers, independent of age, ethnicity, body mass index, lifestyle, years postmenopausal, and site of BMD measurement [8].

The Women’s Health Initiative (WHI), a randomized, double-blind, placebo-controlled prevention trial, evaluated postmenopausal women not selected for low BMD (mean age 63.3 ± 7.1 years). Women with an intact uterus received 0.625 mg/day CEE plus 2.5 mg/day medroxyprogesterone acetate (MPA) (*n* = 8506) or placebo (*n* = 8102). Women with prior hysterectomy received CEE alone (*n* = 5310) or placebo (*n* = 5429). The CEE + MPA trial was terminated early after a median of 5.6 years owing to findings of an increase in breast cancer risk and the unfavorable risk-benefit ratio of this therapy [9]. The CEE-alone trial was terminated after a median of 7.2 years owing to an increase in stroke risk that was not offset by lower coronary heart disease risk [10]. The median cumulative follow-up was 13.2 years for the CEE + MPA trial and 13.0 years for the CEE-alone trial. During the intervention phase, compared with placebo, significant reductions were observed in vertebral fracture in the CEE + MPA trial (HR 0.68; 0.48–0.96), and in the CEE-alone trial (HR 0.64; 0.44–0.93). Significant reductions were observed in all fractures in both trials (the CEE + MPA trial, HR 0.76, 0.69–0.83; the CEE-alone trial, HR 0.72; 0.64–0.80). In the CEE-alone trial, a significant hip fracture benefit in the CEE-alone group versus the placebo group persisted after an average 6.8 years of follow-up (HR 0.61; 0.41–0.91) [10], and after 13.2 years (HR 0.81; 0.68–0.97) [13, 14]. Consistent with the WHI, women in the NORA who were currently on MHT had a 40% lower incidence of hip fractures compared with those who had never used MHT (adjusted OR, 0.60; 0.44–0.82) [11].

The Million Women Study was conducted primarily to examine the health effects of MHT in postmenopausal women aged 50–69 years [12]. This prospective observational questionnaire study demonstrated that compared with never users, current users of MHT at baseline had a significantly reduced incidence of fracture (RR 0.62; 0.58–0.66) [15]. The relative risk of fracture decreased significantly with increasing durations of use. Among current users at baseline, the significant reduction in the relative risk of fracture did not vary significantly according to the personal characteristics of the study participants or whether CEE-only, CEE-progestin, or other types of hormones were used. Past users of hormone therapy at baseline experienced no significant protection against fractures.

According to a meta-analysis [16•] of 28 studies with 33,426 participants and 2516 fractures cases, MHT was associated with a significant reduction in the overall relative risk of total fractures (RR 0.74; 0.69–0.80), hip fractures (RR 0.72; 95% CI 0.53–0.98), and vertebral fractures (RR 0.63; 0.44–0.91). Women younger than 60 years of age had a lower risk of total fractures (RR 0.55; 0.44–0.68) than women > 60 years of age (RR 0.77; 0.71–0.84). Estradiol as well as CEE led to a decrease in the risk of total fractures (RR 0.55; 0.44–0.70, and RR 0.77; 0.71–0.83, respectively) [16•]. Interestingly, a significantly greater BMD gain was observed in women who received CEE plus MPA compared with those who received estrogen alone [17]. However, there are no head-to-head fracture prevention trials of estrogen alone vs. estrogen plus MPA/progesterone.

*Alternative dosages and routes of MHT* have been shown to be efficacious even at low doses. Oral micronized 17β-estradiol at a dose of 0.25 mg/day for 3 years in postmenopausal women resulted in significant increases in hip, spine, and total BMD compared with the placebo. This treatment reduced biochemical markers of bone turnover to a degree comparable with an estrogen dose of 1.0 mg/day. The side effect profile of the drug was similar to that of the placebo [18]. The addition of progestogen to estrogen did not interfere with this benefit.

The effect of low-dose estrogen therapy via the transdermal route on bone preservation has been well documented [19–22]. A double-blind, placebo-controlled study was performed in 355 nonosteoporotic postmenopausal women who had hysterectomies with or without oophorectomy. At 2 years, compared with the baseline values, lumbar spine BMD declined by 0.59% in the placebo group, but it increased by 1.65%, 4.08%, and 4.82% in the estradiol 0.025, 0.05, and 0.075 mg/day groups, respectively [23]. Administration of 0.050 mg/day or 0.025 mg/day transdermal estradiol resulted in a reduction in bone turnover markers to a similar degree [19]. Transdermal administration of 0.014 mg/day estradiol was associated with a significant increase in lumbar spine BMD and in total hip BMD compared with the placebo group [20]. A 2-year transdermal administration of estradiol and levonorgestrel resulted in significant increases in the lumbar spine, hip, and total body BMD, in the hormone groups compared with the placebo group [21]. All bone markers were significantly reduced in the hormone groups compared with the placebo group [21]. According to a meta-analysis of nine clinical trials, lumbar spine BMD increased significantly by 3.4% and 3.7% after one and 2 years of transdermal estrogen therapy, respectively, compared with the baseline values [22].

Effects of intranasal estradiol (pulsed estrogen therapy) was demonstrated in 386 women, less than 5 years past menopause, randomized to intranasal placebo or estradiol at a dose of 150 μg or 300 μg daily for 2 years. Women with an intact uterus, treated with estradiol, received 200 mg micronized progesterone per day for 14 days of each 28-day cycle. Compared with placebo, BMD increased significantly by 5.2% and 6.7% at the spine and 3.2% and 4.7% at the hip as a result of treatment with 150 μg and 300 μg estradiol, respectively [24]. Serum markers of bone remodeling decreased to premenopausal levels in the treated groups. At least one adverse event (rhinitis, sneezing, and application site reaction) was reported in 94% of treated women [24].

*The effects of discontinuation of estrogen use* on the rate of bone loss was demonstrated in observational studies [25], in clinical trials [26, 27], and in a prospective study among past hormone users compared with current users [8]. Postintervention, the fracture risk reductions were attenuated in both WHI trials, but a significant fracture benefit persisted after 13 years for CEE + MPA (HR 0.81; 0.68–0.97). In the NORA study, women who stopped using MHT more than 5 years earlier had a similar hip fracture risk as that of never users [11]. In a follow-up of the prospective epidemiological study focusing on risk factors for osteoporosis and cardiovascular disease (the PERF study), 263 healthy postmenopausal women who received either MHT or placebo for 2–3 years with no further bone-sparing treatment until follow-up were reexamined 5, 11, or 15 years after stopping MHT [28•]. The risk of all osteoporotic fractures (OR 0.48; 0.26–0.88) and vertebral fractures (OR 0.47, 0.24–0.93) was significantly reduced compared with the placebo-treated women [28•].

## Bone Effects of Estrogen Deficiency

After menopause, bone homeostasis is dysregulated by hormonal deficiency, leading to enhanced bone resorption and, consequently, increased bone formation. However, the rate of formation is not able to keep up with the rate of resorption, resulting in net bone loss [29, 30]. Estrogen deficiency plays a specific role in the subclinical inflammatory bone-microenvironment state that is accompanied by an increase in oxidative stress and the generation of advanced glycation end products [31, 32•]. Estrogen deficiency in early postmenopausal women is characterized by progressive osteoclastic hyperactivity associated with an increase in serum concentrations of proinflammatory cytokines (TNF-α, IL-1, IL-6, and IL-17) and CRP [33–35]; upregulation of RANKL, M-CSF, and M-CSF receptor; and decreased expression of osteoprotegerin [36]. IL-17 levels are significantly elevated in postmenopausal osteopenic or osteoporotic women compared with those in premenopausal women and are positively correlated with the sRANK ligand or the ratio of the sRANK ligand to OPG [37]. The RANK/RANKL/OPG axis is also actively regulated by B cells [38]. The surface concentration of RANKL of the marrow cells, characterized as osteoblast lineage, T cells, or B cells, was increased in estrogen-deficient postmenopausal women compared with premenopausal women. These increases were reversed by estrogen treatment [36]. The relative decrease in bone formation after menopause may be explained by increased sclerostin levels, differentiation of osteoblast progenitors toward the adipocyte lineage, and osteoblast apoptosis induced by products of oxidative stress, including reactive oxygen species [39, 40].

Experimentally, the mechanism by which hormonal changes are associated with increased numbers of T cells or macrophage activation was suggested to be a permissive connection between estrogen deficiency, the gut microbiota, inflammation, and TNF-α production [41]. The gut microbiota is a potent modulator of the immune system and numerous other physiological processes, both in the gut and throughout the whole organism. Disruption of gut microbiota is a hallmark of many inflammatory, neurological, neoplastic and metabolic diseases. The gut microbiota achieves this extraordinarily broad scope of effects partly directly, by producing bioactive metabolites, and partly indirectly, by shaping the cellular and humoral response of the host’s cells, mainly in the gut epithelial and immune cells [42]. The overall outcome is a result of multiple mechanisms, including preosteoclast proliferation, differentiation and apoptosis, being triggered or blocked by one or several of these cytokines [43]. Maintaining the gut barrier is one of the major functions of the mucosa and its failure is associated with chronic inflammatory diseases, often with the contribution of adherent or invasive microbes [44]. Damage to the gut barrier may lead to the excessive stimulation of the immune system, thus worsening chronic inflammation and barrier function [45]. Intestinal epithelial cells contain estrogen receptors capable of regulating gene transcription. Experimentally, estrogen deficiency was associated with increased gut permeability, an expansion of Th17-activated T cells, and subsequent upregulation of the osteoclastogenic cytokines TNF-a, RANKL, and IL-17. T-follicular helper cells, along with B cells, participate in the formation of germinal centers and antibody production [46]. Estrogens may augment gap junctions and cell-to-cell contact, as well as promote changes in the composition and diversity of the gut microbiota, which is associated with immune regulation [47, 48].

## Bone Effects of Menopausal Hormone Therapy

The results of in vitro experiments indicated that estrogens suppress RANKL production in T and B cells [36] and significantly reduce the recruitment and increase apoptosis of osteoclasts [49, 50]. Estrogen replacement reverses the release of TNF-α and IL-1 from peripheral blood monocytes in women after natural or surgical menopause [33, 51]. After acute estrogen withdrawal in postmenopausal women, the increase in bone resorption was significantly blocked by the TNF-a blocker and to a lesser extent by the IL-1b blocker. However, the markers of bone formation were not significantly reduced [52]. Estrogens increase osteoblast survival and type 1 collagen production [53]. Accordingly, estrogens exert an anabolic effect on bone in cell and tissue studies and in animal models. Estrogens regulate the production of the inhibitor of Wnt signaling, sclerostin [54]. In mice, the sclerostin-Fc vaccination reduced serum sclerostin concentrations, increased serum levels of bone formation markers, alleviated the ovariectomy-induced increase in serum resorption markers, and improved the estrogen deficiency-mediated deterioration of BMD, morphometric characteristics of trabecular bone, and mechanical strength of the femur and lumbar spine [55]. Treatment of postmenopausal women with estrogen or raloxifene reduces circulating and bone marrow plasma levels of sclerostin as well as bone sclerostin messenger RNA (mRNA) [56]. In a study with higher than conventional doses of estrogen replacement (75 mg implant estradiol every 6 months) in postmenopausal women (mean age 65.6 years), BMD improved in every patient, with a median increase of 31.4% at the lumbar spine and 15.1% at the proximal femur [57]. Bone histomorphometry showed a significant increase in cancellous bone volume and wall thickness, indicating net bone gain. A significant positive correlation was found between serum estradiol and posttherapy cortical collagen and between an increase in lumbar spine BMD and cortical collagen content. An increase in collagen turnover rather than just the accumulation of mature collagen with increasing age was supported by an increase in intermediate crosslinks in both cortical and cancellous bone and in mature crosslinks in cortical bone [57].

Important information regarding the effects of MHT in the preservation of bone mass by reducing the rate of osteoclastic bone resorption and maintaining new bone formation was provided by dynamic bone histomorphometry in a randomized, double-blind, clinical prospective trial that enrolled healthy women aged 45-55 years who were treated for 2 years with either cyclic estradiol/norethisterone acetate or placebo [58•]. Bone biopsies from untreated women demonstrated an increased osteoclastic erosion rate, erosion surface and erosion depth compared with women in the MHT group. In untreated women, delayed osteoclast apoptosis resulted in a longer osteoclast lifespan and increased resorptive activity and erosion depth. The bones of women taking MHT were characterized by preservation of bone balance at individual basic multicellular units (BMUs) (wall thickness-erosion depth) and no change in erosion depth or osteoclastic erosion depth. A relative osteoblastic insufficiency was present in the placebo group because osteoblastic bone formation was unable to keep up with the increase in bone resorption [58•]. Accordingly, a significant decrease in osteoclast number and osteoclastic resorption rate, but not mineralizing surface or bone formation rate, was observed after 6 months of CEE treatment [59].

Both MHT and antiresorptive therapies such as aminobisphosphonates and denosumab increase BMD, reduce bone turnover and are efficacious in the treatment of postmenopausal osteoporosis [60–62]. A reduction in the number of remodeling sites may decrease the probability of trabecular perforation and failure and thus stabilize the trabecular network [63]. However, estrogen but not antiresorptive therapies [64–67], by enabling maintenance of bone formation, can adjust the physiological rate of bone remodeling, and restore quality of bone organic matrix, that affects bone micromechanical properties independently of mineralization [68–72]. Moreover, estrogen but not antiresorptive therapies [73] attenuate the inflammatory bone-microenvironment and maintain the equilibrium between bone resorption and bone formation by modulating osteoblast/osteocyte and T cell regulation of osteoclasts.

Of importance are beneficial effects of MHT on connective tissue, namely, muscle and cartilage. In a meta-analysis, postmenopausal women treated with MHT had approximately 5% greater muscle strength than those not on MHT [74]. According to Collins et al. the loss of muscle strength in females resulting from estrogen deficiency appears to be associated with apoptotic pathways that contribute to the loss of muscle mass, inadequate preservation of skeletal muscle mass and reduced quality of the remaining skeletal muscle [75•]. Estrogen may protect skeletal muscle against apoptosis via its effects on hydrogen peroxide-induced apoptosis and mitochondrial dysfunction [75•]. On the other hand, the cross-sectional area of the skeletal muscles around the femur was lower in osteoporotic patients who underwent long-term aminobisphosphonate treatment than that of the BMD-matched control postmenopausal women [76].

A significantly lower intervertebral disc height was demonstrated in postmenopausal osteoporotic women when compared with that in untreated nonosteoporotic women who, in turn, had significantly lower disc height than premenopausal women and women taking MHT [77]. Intervertebral disc space shows a progressive decline that almost entirely occurs in the first 5–10 years after menopause [78]. Estrogen was shown to have direct chondroprotective effects and to be able counteract cartilage degradation in an in vivo model of increased cartilage turnover [79].

## Safety Aspects of Menopausal Hormone Therapy

According to clinical studies [13, 80], the individual benefit/risk balance of MHT is very dependent on the type, doses, and duration of MHT as well as the individual risk profile of each woman. These aspects are considered in the guidelines [80–84••].

In women younger than 60 years in the WHI trial, after 13 years of treatment with CEE alone, relative risks were decreased for breast cancer (RR 0.76; 0.52–11) and all cancers (RR 0.80; 0.64–0.99) [85]. However, in the CEE-alone WHI trial, the risk of ER+/PR+ breast cancers was noted to be significant after 15 years of current CEE use (RR 1.48; 1.05–2.07) [86]. Therapy with CEE plus MPA for a median of 5.6 years or with CEE alone for a median of 7.2 years was not associated with risk of cancer mortality during a cumulative follow-up of 18 years [87••]. However, in the CEE + MPA WHI trial, breast cancer risk steadily increased throughout the intervention, and was significantly increased for the entire intervention phase (HR 1.24; 1.01-1.53) [88]. The risk of breast cancer appears to be increased with the increasing duration of use. According to Santen and Yue, an average of 16 years is required for tumors to undergo the 30 doubling times necessary for the occult tumors to reach the threshold for clinical detection [89•]. Thus, CEE plus a progestogen in the WHI study increased the rate of tumor diagnosis [89•]. Recent individual participant meta-analysis of the worldwide epidemiological evidence from eligible prospective studies of MHT use in 108,647 postmenopausal women who developed breast cancer at mean age 65 years, has concluded that every MHT type, except vaginal estrogens, was associated with excess breast cancer risks, which increased steadily with duration of use [90••]. Breast cancer risks were greater for estrogen-progestagen than estrogen only preparations. Among current users, these excess risks were definite even during second to fourth year (estrogen-progestagen RR 1.60, 95% CI 1.52–1.69; estrogen-only RR 1.17, 1.10–1.26) and were twice as great during years 5–14 (estrogen/progestagen RR 2.08, 2.02–2.15; estrogen-only RR 1.33, 1.28–1.37). According to this meta-analysis, 5 years of MHT, starting at age 50 years, would increase breast cancer incidence at ages 50–69 years by about one in every 50 users of estrogen plus daily progestagen preparations; one in every 70 users of estrogen plus intermittent progestagen preparations; and one in every 200 users of estrogenonly preparations. The corresponding excesses from 10 years of MHT would be about twice as great.

The risk of breast cancer was found to be influenced by the type of MHT used [91]. A prospective, longitudinal cohort study of BRCA1 and BRCA2 mutation carriers indicated that after 10 years of follow-up, the cumulative incidence of breast cancer among women who used estrogen-alone MHT was 12% compared with 22% among women who used estrogen plus progesterone MHT (*p* = 0.04) [92]. A meta-analysis of 14 studies of women using estradiol with or without progestogen showed no association between estradiol only and risk of breast cancer; however, the risk was significantly elevated when estradiol was combined with MPA, norethisterone acetate, or levonorgestrel but not when it was combined with progesterone or dydrogesterone [93]. Compared with a conventional dose of CEE, transdermal estrogen was associated with a nonsignificant lower risk of invasive breast cancer (HR 0.75; 0.47 -1.19) [94•].

Importantly, in women younger than 60 years in the WHI trial, after 13 years of treatment with CEE alone, relative risks were decreased for coronary heart disease (RR 0.65; 0.44–0.96), myocardial infarction (RR 0.60; 0.39–0.91), and all-cause mortality (RR 0.78; 0.59–1.03) [85]. Treatment with CEE plus MPA for a median of 5.6 years or with CEE alone for a median of 7.2 years was not associated with risk of all-cause or cardiovascular mortality during a cumulative follow-up of 18 years [87••]. According to results of a meta-analysis of 19 trials, women who started MHT less than 10 years after menopause or who were less than 60 years old, had significantly lower all-cause mortality (RR 0.70; 0.52–0.95) and coronary heart disease (RR 0.52; 0.29–0.96) compared with the placebo population [95]. However, this meta-analysis demonstrated a nonsignificant trend toward risk of stroke (RR 1.37; 0.80–2.34), and significant increase in the risk of venous thromboembolism (VTE) (RR 1.74; 1.11–2.73) [95] A meta-analysis of effects of oral versus transdermal estrogen indicated that among women using estrogen-only preparations, oral but not transdermal preparations increased VTE risk (RR 1.48, 1.39–1.58; RR 0.97, 95% CI 0.87–1.09, respectively) [96•]. In transdermal estrogen users, there was no change in VTE risk in women using micronized progesterone (RR 0.93, 0.65–1.33), whereas norpregnane derivatives were associated with increased VTE risk (RR 2.42, 1.84–3.18). Among women using opposed oral estrogen, there was a higher VTE risk in women using medroxyprogesterone acetate than in those using other progestins [96•].

Ischemic stroke is affected differentially by the route of estrogen administration [16•] due to the hepatic first-pass effect of estrogens when administered orally. Oral but not transdermal estrogen activates the coagulation cascade and increases fibrinolytic activity and may also induce resistance to activated protein C, which has been associated with an increased VTE risk [97. 98].

Other risks associated with MHT include dementia (in women aged ≥ 60 years) [99], gallbladder disease [100], and urinary incontinence [101].

## Conclusion

MHT prevents bone loss and deterioration of the bone microarchitecture [102] and decreases the incidence of osteoporosis-related fractures even in postmenopausal women not diagnosed with osteoporosis, with an efficacy similar to that of bisphosphonates. However, due to differences in the mode of action, estrogen but not antiresorptive therapies can attenuate the inflammatory bone-microenvironment and maintain the physiological equilibrium between bone resorption and bone formation. Importantly, the use of MHT for 5-10 years from the onset of menopause has potentially valuable effects on the bone for many years after MHT discontinuation [16•, 28•]. Also, MHT during the early postmenopausal years effectively improveshot flashes and night sweats, and may improve other features involved in the genesis of osteoporotic vertebral fractures, namely, the quality of connective tissue.

The evidence of increased risks of breast cancer does not allow recommending MHT for the prevention of bone loss in the population, even in women younger than 60 years old or who are within 10 years of menopause onset. However, MHT should be considered in women with premature estrogen deficiency and increased risk of bone loss and osteoporotic fractures. Selective estrogen receptor modulators [103–106] appear to be an available option to delay the necessity of antiresorptive therapies in postmenopausal women as they age. Clinical trials are needed to test the efficacy, safety and cost-effectiveness of other antiresorptive options in the prevention of accelerated bone loss in the early years after menopause in women with increased risk of an accelerated bone loss in order to reduce the number of future fractures associated with the changing population demography.
